# Bullying among adolescents in a Brazilian urban center – “Health in Beagá” Study

**DOI:** 10.1590/S0034-8910.2015049005188

**Published:** 2015-09-10

**Authors:** Michelle Ralil da Costa, César Coelho Xavier, Amanda Cristina de Souza Andrade, Fernando Augusto Proietti, Waleska Teixeira Caiaffa

**Affiliations:** I Faculdade de Medicina. Universidade Federal de Minas Gerais. Belo Horizonte, MG, Brasil; II Faculdade da Saúde e Ecologia Humana. Vespasiano, MG, Brasil

## Abstract

**OBJECTIVE:**

To analyze the prevalence of bullying and its associated factors in Brazilian adolescents.

**METHODS:**

Data were used from a population-based household survey conducted by the Urban Health Observatory (OSUBH) utilizing probability sampling in three stages: census tracts, residences, and individuals. The survey included 598 adolescents (14-17 years old) who responded questions on bullying, sociodemographic characteristics, health-risk behaviors, educational well-being, family structure, physical activity, markers of nutritional habits, and subjective well-being (body image, personal satisfaction, and satisfaction with their present and future life). Univariate and multivariate analysis was done using robust Poisson regression.

**RESULTS:**

The prevalence of bullying was 26.2% (28.0% among males, 24.0% among females). The location of most bullying cases was at or on route to school (70.5%), followed by on the streets (28.5%), at home (9.8%), while practicing sports (7.3%), at parties (4.6%), at work (1.7%), and at other locations (1.6%). Reports of bullying were associated with life dissatisfaction, difficulty relating to parents, involvement in fights with peers and insecurity in the neighborhood.

**CONCLUSIONS:**

A high prevalence of bullying among participating adolescents was found, and the school serves as the main bullying location, although other sites such as home, parties and workplace were also reported. Characteristics regarding self-perception and adolescent perceptions of their environment were also associated with bullying, thus advancing the knowledge of this type of violence, especially in urban centers of developing countries.

## Abstract (pt)

**OBJETIVO:**

Analisar a prevalência e fatores associados ao *bullying* em adolescentes brasileiros.

**MÉTODOS:**

Estudo populacional com 598 adolescentes de 14 a 17 anos da área urbana de Belo Horizonte em 2009. Foram utilizados dados de um inquérito domiciliar realizado pelo Observatório de Saúde Urbana, com amostragem probabilística em três estágios: setores censitários, residência e indivíduos. Os adolescentes responderam questões sobre *bullying*, características sociodemográficas, comportamentos de risco à saúde, bem-estar educacional, estrutura familiar, atividade física, marcadores de hábitos alimentares e bem-estar subjetivo (percepção corporal, satisfação pessoal e satisfação com a vida atual e futura). Foram realizadas análises univariadas e múltiplas por meio do modelo de Poisson robusto.

**RESULTADOS:**

A prevalência de *bullying* foi de 26,4% (28,0% no sexo masculino, 24,0% no sexo feminino). O b*ullying* associou-se com insatisfação com a vida, dificuldades de relacionamento com os pais, envolvimento em brigas com pares e insegurança na vizinhança. O local de maior ocorrência foi a escola ou percurso escolar (70,5%), seguido por rua (28,5%), domicílio (9,8%), praticando esporte (7,3%), festa (4,6%), trabalho (1,7%) e outros locais (1,67%).

**CONCLUSÕES:**

Há alta prevalência de *bullying* entre os adolescentes investigados, e a escola é apontada como principal local de ocorrência, embora locais como domicílio, festa e trabalho também sejam relatados. A percepção que o indivíduo tem de si e do seu contexto associa-se ao *bullying*, avançando, portanto, no conhecimento deste tipo de violência especialmente em centros urbanos de países em desenvolvimento.

## INTRODUCTION

Bullying is an aggressive behavior that can be defined as “being the tough guy against someone”. The meaning of the term bullying, which does not have an appropriate translation in Portuguese, can vary according to the culture. A study on the meaning of the term in 14 countries pointed to important differences between languages and even within the same language.^20^ It generally refers to an affirmation of interpersonal power in which the aggressor is referred to as the bully, which in Portuguese is translated as “tough guy”, and the target of the aggression is the victim. The dynamics of this type of violence is characterized by an imbalance of power and a lack of reciprocity.^a^

Although the term is used to name the practice of systematic humiliation of children and adolescents especially in the school environment, bullying surpasses the school walls. Homes, workplaces and even prisons are sites of bullying, despite the popular image of bullying as a school-based phenomenon.^19^

Bullying can be considered a health problem resulting in behavioral and emotional issues for the victims, including stress, decreased self-esteem, anxiety, depression, poor academic performance, and, in severe cases, even death.^3^ Bullying and its consequences are directly related to well-being and has been the subject of various studies, including a report presented by UNICEF in February 2007, addressing a global vision of child well-being in 21 developed countries.^b^

In a major study from 2007, 1/3 of children in the 21 European countries composing the Organization for Economic Co-operation and Development (OECD) claimed to have been bullied at least once during the two previous months, and a similar proportion of children declared to bully other children.^b^ The prevalence of students victimized varies between 8.0% and 46.0%, and of aggressors varies between 5.0% and 39.0%, with 20.0% of the children into both categories.^b^ ^,^ ^c^ ^,^ ^d^

The National Study on School Health (PeNSE), the most comprehensive study on the topic in Brazil, conducted in 2009, the prevalence of victimization by bullying found in Brazilian state capitals was 30.8%, with the highest frequency (35.3%) being found in Belo Horizonte, Southeastern Brazil.^e^ In the same study, the prevalence of victimization by bullying was 35.4% in 2012.^f^

**Figure 1:**
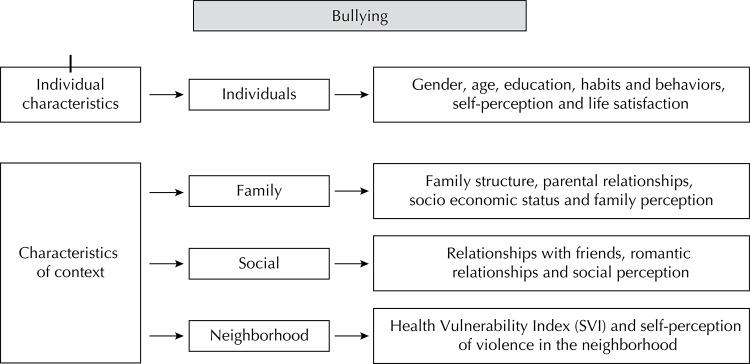
Theoretical model proposed for this study, based on the Urban Health Model adapted from Galea & Vlahov,11 2005, by Caiaffa et al,4 2008.

Many factors are associated to bullying (including both profiles: aggressor and victim), such as: age,^17^ family dynamics,^19^ interpersonal relationships,^9^ social context, consumption of alcohol and tobacco, poor academic performance, feelings of despair, loneliness, and quality of life should be highlighted.^1^ ^,^ ^18^ Bullying can be influenced by proximal and distal factors, such as individual and family, social and neighborhood characteristics, respectively^4^ (Figure 1).

National and international studies have generally approached bullying using school surveys. Although factors associated with bullying have been identified, most studies have been undertaken in developed countries, thus these factors may not coincide with the Brazilian reality. Furthermore, population-based studies can contribute to bullying research.

This study aimed to analyze the prevalence of bullying and its associated factors in Brazilian adolescents.

## METHODS

In this cross-sectional study, we analyzed data from the “Beagá Health Study”,^10^ a household survey undertaken in two sanitary districts of Belo Horizonte, MG, Southeastern Brazil, in 2009, representing 24.0% of the city population of 2,375,151 inhabitants,^g^ and with a large socioeconomic heterogeneity.

The sampling was stratified in three stages. The stratification factor was the health vulnerability index (IVS in the Portuguese acronym), which was determined by combining social, demographic, economic, and health indicators. At each level, the following were randomly selected: census tract; household; and individual, where once in the household, an adult resident (> 18 years old) and, if existing, an adolescent (between 11 and 17 years) were selected. From a frame list provided by the municipality, 149 census tracts were randomly selected in the two sanitary districts of the city. Vacant lots, institutional and commercial buildings, vacant households or those that were closed after three tentative visits were deleted. Within census tract, 5,171 households comprised the eligible list, deriving a study sample of 4,048 residents ≥ 18 years and 1,197 adolescents. Due to 13.3% of refusals, incomplete interviews, and lost of contact after three attempts, the final sample of adolescents ranging from 11 to 17 years old consisted of 1,048 participants. Out of them, this study refers to 598 adolescents aged from 14 to 17 years who answered the specific risk behavior questionnaire including bullying questions.

The questionnaire was self-applied and included questions about: physical activity; sexual initiation and risk behaviors such as consumption of alcohol, drugs, and tobacco; and involvement in violent situations, including bullying. The collection instruments were prepared by OSUBH based on Brazilian,^e^ and international^b^ ^,^ ^h^ studies. All instruments were pre-coded and pre-tested in a pilot study.

The dependent variable (bullying) was obtained by the question “Have you suffered from repeated intimidation, offense, aggression or harassment that made you feel humiliated or afraid?”, which investigated bullying from the perspective of the victim. If so, “Where did this take place?”. The participant could select multiple answers to better understand the locations of bullying.

From the perspective of the theoretical model proposed by this study (Figure 1), based on the Urban Health Model from Galea & Vlahov^11^ and adapted by Caiaffa et al,^4^ the independent variables represented the following proximal factors: individual variables (age, gender, student status during the study year, type of school [public; private], grade repetition, consumption of tobacco, alcohol or illicit drugs, body image, mental well-being, and life satisfaction); and the following distal factors: family-related variables (educational attainment of the head of household, family conversations, if the family eats meals together, reports of family conflicts, adolescent perception of being loved, parental supported, being able to share problems with parents, reports of fighting with parents, sufficient parental attention or involvement, and if parents cause adolescents to feel badly about themselves); perceptions of the social environment (whether friends are helpful and fun, feelings of exclusion, feelings of awkwardness or discomfort in social settings, feelings of loneliness), and variables related to the neighborhood (measured by the IVS, a geocoded health risk indicator based on place of residence, and insecurity within the neighborhood as perceived by the adolescent).

The “Faces Scale”^2^ and the “Life Satisfaction Scale”^6^ were used to evaluate psychological well-being. Body satisfaction was evaluated by a scale developed to the Brazilian population.^12^

The household head’s educational level questions were answered by an adult resident and categorized by years of study (≤ 8 years and > 8 years).

Prevalence rates and corresponding 95% confidence intervals were calculated for bullying in the categories proposed. The variable (bullying) was dichotomized (yes; no) and described according to the domains of the proposed theoretical model (individual, social, familiar, and neighborhood characteristics). Pearson’s Chi-squared test was used to evaluate the association between bullying and the variables.

Unadjusted and adjusted analyses were done using prevalence ratios (PR) estimated according to the Poisson model with robust variance. The independent variables with a p < 0.20 in the univariate analysis were candidates for multiple analysis, along with other epidemiologically relevant variables.

The final model was controlled for gender and age to minimize the effects of these characteristics on the other variables included in this study. Variables with p ≤ 0.05 remained in the final model. The appropriateness of the model was evaluated by the deviance test.

Stata software (Stata Corp., College Station, USA) version 10.0 for complex samples was used due to the sample weight and design.^i^

This study was approved by the Research Ethics Committees of the Universidade Federal de Minas Gerais and the Municipal Health Department of Belo Horizonte (Process ETIC 253/03 – Extension 01/08). The informed consent form was signed by the adolescents and their parents.

## RESULTS

The sample included 598 adolescents aged from 14 to 17 years. Most adolescents were males (53.0%) and students (92.9%), of which 82.9% studied in public schools (Table 1).

**Table 1:** Distribution of individual characteristics of adolescents (14 to 17 years) victims of bullying. Beagá Health Study, Belo Horizonte, MG, Southeastern Brazil, 2008 to 2009.

Variable	Total		Reports of bullying		p^b^

	n	%^a^	%^a^	95%CI	
Individual – Individual characteristics					
Gender					
Feminine	285	46.6	28.0	22.1;33.9	0.393
Masculine	313	53.4	24.2	18.2;30.2	
Age (years)					
14	154	27.9	22.2	14.1;30.4	0.196
15	172	27.5	23.8	16.3;31.2	
16	130	21.3	25.8	16.6;34.9	
17	142	23.3	34.4	25.6;43.2	
Currently studying (in 2008)					
Yes	549	92.9	26.7	22.3;31.2	0.399
No	49	7.1	19.3	4.8;33.9	
Type of school					
Public	460	77.0	27.0	22.1;31.8	0.571
Private	86	15.3	26.5	15.6;37.5	
Not studying/Other	52	7.7	18.0	4.3;31.7	
Have you repeated a grade?					
Yes	229	42.0	26.0	19.2;32.7	0.900
No	351	58.0	26.5	20.9;32.2	
In the past year, have you been involved in a fight in which someone has gotten hurt?					
Yes	77	14.6	45.1	31.9;58.4	**0.003**
No	517	85.4	23.5	19.0;27.1	
Have you ever used drugs?					
Yes, at least once	42	6.9	43.2	24.1;62.4	**0.044**
Never	553	93.1	25.0	20.8;29.3	
Do you currently drink alcohol?					
Yes	73	11.9	35.7	23.2;48.1	0.085
No	525	88.1	24.9	20.6;29.3	
Have you been drunk?					
At least once	121	19.0	40.6	30.3;51.0	**0.001**
Never	477	81.0	22.8	18.3;27.3	
Do you currently smoke?					
Yes, daily/Sometimes	20	3.4	51.3	22.9;79.7	**0.045**
No	578	96.6	25.3	21.2;29.5	
Psychological well-being					
Distress	28	4.3	43.6	21.9;65.3	0.069
Well-being	570	95.7	25.4	21.3;29.6	
Life satisfaction					
Dissatisfied	81	14.1	44.9	31.5;58.3	**0.001**
Satisfied	517	85.9	23.2	19.1;27.2	
Body image					
Dissatisfied	464	80.7	28.6	23.5;33.7	**0.019**
Satisfied	120	19.3	16.4	9.1;23.6	

a Prevalence considering the sample weight and size.

b Chi-square test.

Values with statistical significance are shown in bold.

**Figure 2:**
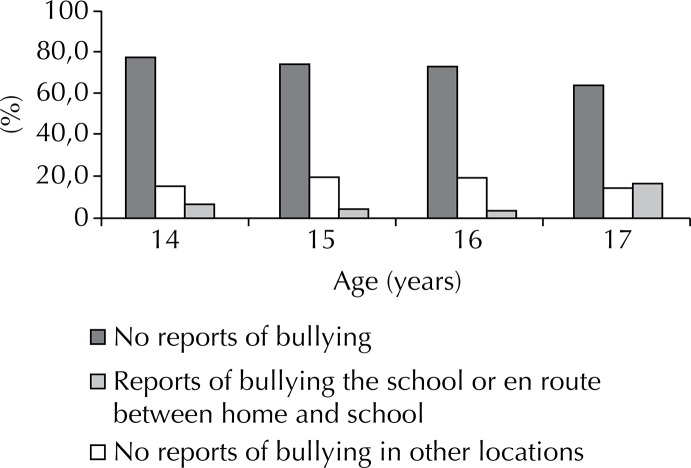
Bullying according to age. Beagá Health Study, Belo Horizonte, MG, Southeastern Brazil, 2008 to 2009.

The prevalence of reported bullying was 26.4% and the proportion did not differ according to age or gender. The location with the greatest frequency of bullying was the school (55.1%) followed by on the streets (28.5%), en route between home and school (15.4%), home (9.8%), practicing sports (7.3%), at parties (4.6%), at work (1.7%), and at other locations (1.6%). Reports of bullying in the school environment (at school or en route between school and home, which together account for 70.4%), did not differ between ages, while reports at other locations were most prominent among those with 17 years old (Figure 2).

Reports of bullying were more frequent among adolescents who were in fights in which someone got hurt within the last year, who have used drugs at least once, who have been drunk at least once, and who were smokers at the time of the survey. The prevalence of bullying was higher among those who reported worse perceptions of their psychological well-being, such as psychological distress and negative levels of life satisfaction. Similar results were found among those who reported being dissatisfied with their body image (Table 1).

**Figure 3:**
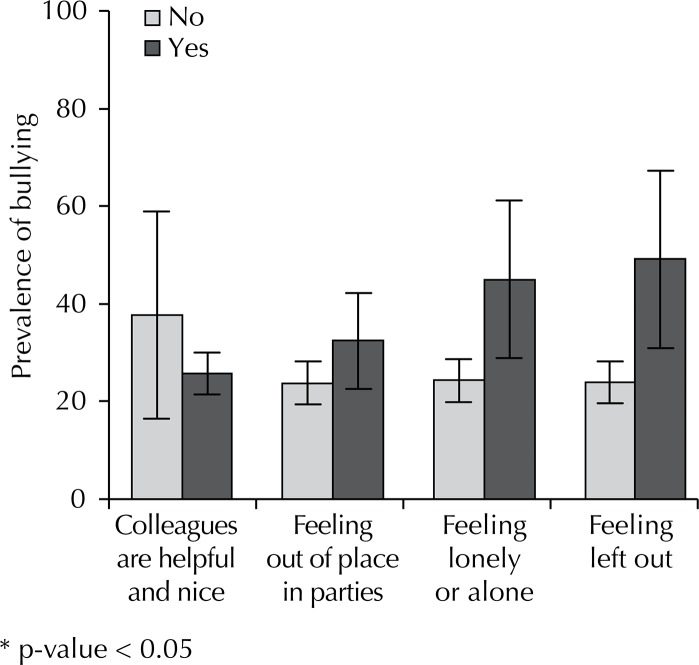
Prevalence of bullying according to social characteristics. Beagá Health Study, Belo Horizonte, MG, Southeastern Brazil, 2008 to 2009.

The prevalence of bullying did not differ depending on whether friends were perceived as helpful and fun or if the participant felt awkward or uncomfortable at parties (Figure 3). However, bullying was more frequent among adolescents who felt excluded or alone. The strong association between the last two variables and bullying might be attributed to the conceptual proximity of these variables within this model. For this reason, it was decided to not include feelings of exclusion and feelings of loneliness or being alone in the adjusted analysis.

Pronounced collinearities were found between the variables bullying and risk behaviors (participating in fights, drug-use, consumption of alcohol or tobacco, episodes of drunkenness). It was decided to only include “having participated in a fight in which someone got hurt within the last year” in the final model.

Reports of bullying were more frequent among adolescents whose head of household had higher educational level and among adolescents with negative perceptions of their families, including: few conversations with parents, many fights within the family, impossibility of discussing problems with parents, many fights with parents, and insufficient parental attention. Bullying generally decreased when vulnerability increased: 32.4% of the adolescents who were victim of bullying lived in areas of low vulnerability, 26.2% in areas of moderate vulnerability, 25.3% in areas of high vulnerability, and 22.8% in areas of extreme vulnerability (Table 2).

**Table 2:** Distribution of contextual characteristics of adolescents (14 to 17 years) victims of bullying. Beagá Health Study, Belo Horizonte, MG, Southeastern Brazil, 2008 to 2009.

Variable	Total		Reports of bullying		p^b^

	n	%^a^	%^a^	95%CI	
Contextual – Family characteristics					
Educational attainment of head of household (years)					
0 to 8	339	56.2	20.6	15.5;25.6	**0.004**
9 or more	251	43.8	33.2	26.5;40.0	
How often do you speak with your parents?					
Always	338	54.8	21.4	16.5;26.2	**0.012**
Never/Rarely/Sometimes	259	45.2	32.2	25.1;39.4	
Do you eat your meals with your parents?					
Never	54	9.8	34.6	17.7;51.5	0.242
Sometimes	542	90.2	25.2	21.1;29.4	
In comparison with other families you know, are there fights in your family?					
Many fights	74	26.8	43.6	30.5;56.8	**0.006**
Few fights	360	60.4	24.9	19.2;30.5	
No fights	163	12.8	19.6	12.0;27.2	
My parents make me feel loved and cared for					
Yes	572	96.7	25.7	21.5;29.9	0.128
No	20	3.3	45.6	16.7;74.5	
My parents are always on my side					
Yes	565	95.6	26.1	21.8;30.3	0.306
No	27	4.4	37.9	13.6;62.1	
I can discuss problems with my parents					
Yes	477	79.4	21.8	17.5;26.1	**< 0.001**
No	114	20.6	44.1	33.4;54.8	
My parents and I fight a lot					
Yes	84	13.6	39.5	27.9;51.0	**0.008**
No	506	86.4	24.4	20.0;28.7	
My parents give me sufficient attention					
Yes	530	90.0	24.6	20.4;28.9	**0.022**
No	63	10.0	40.8	25.9;55.7	
My parents make me feel badly about myself					
Yes	43	6.8	40.1	22.6;57.6	0.063
No	548	93.2	25.4	21.3;29.5	
Type of family					
Traditional/Nuclear	397	68.5	25.7	20.6;30.7	0.877
Blended/Others	71	12.2	25.9	13.9;37.8	
Single parent	130	19.1	28.3	19.6;37.0	
Contextual – Neighborhood characteristics					
Health Vulnerability Index					
Low	69	11.1	32.4	20.7;44.2	0.668
Medium	220	40.7	26.2	19.3;33.1	
High	238	37.9	25.3	18.6;32.0	
Very high	71	10.3	22.8	12.9;32.7	
Do you feel insecure in your neighborhood?					
Yes	275	46.6	31.2	24.1;38.3	**0.044**
No	322	53.4	21.9	16.8;27.1	

a Prevalence considering the sample weight and size.

b Chi-square test.

Values with statistical significance are shown in bold.

Age and gender were used as adjustment variables in the final model due to their epidemiological importance, although no statistical association was found in the univariate analysis. After adjustment, the variables that remained associated with bullying were: reports of fights within the last year, life dissatisfaction, difficulty in discussing problems with parents, educational attainment greater than eight years for the head of household and feelings of insecurity in the neighborhood (Table 3).

**Table 3:** Adjusted and unadjusted prevalence ratios for the association between bullying and individual and contextual characteristics. Beagá Health Study, Belo Horizonte, MG, Southeastern Brazil, 2008 to 2009.

Variable	Unadjusted analysis			Adjusted analysis^a,b^		

	PR	95%CI	p	PR	95%CI	p
Individual – Individual characteristics						
In the past year, have you been involved in a fight in which someone has gotten hurt?						
Yes	1.95	1.41;2.71	**< 0.001**	1.49	1.05;2.12	**0.027**
No	1.00			1.00		
Life satisfaction						
Dissatisfied	1.94	1.38;2.71	**< 0.001**	1.77	1.27;2.47	**0.001**
Satisfied	1.00			1.00		

Contextual – Family and neighborhood characteristics						

I can discuss problems with my parents						
No	2.02	1.49;2.75	**< 0.001**	1.85	1.37;2.50	**< 0.001**
Yes	1.00			1.00		
Educational attainment of head of household (years)						
> 8	1.62	1.17;2.22	**0.003**	1.71	1.26;2.31	**0.001**
≤ 8	1.00			1.00		
Do you feel insecure in your neighborhood?						
Yes	1.42	1.01;1.99	**0.041**	1.41	1.02;1.94	**0.038**
No	1.00			1.00		

PR: prevalence ratio

a Adjusted for gender and age.

b Goodness of fit test (Deviance) – p = 0.812.

Values with statistical significance are shown in bold.

## DISCUSSION

Approximately 1/4 of the adolescents reported being victims of bullying, with boys experiencing a slightly higher frequency than girls. Most adolescents reported the school environment (school or en route between home and school) as the location of bullying. Reports of bullying were associated with involvement in fights with peers, life dissatisfaction, difficulty relating to parents, and neighborhood insecurity.

The prevalence of bullying in this study (26.4%) was lower than that found by the National School Health Survey (PeNSE) conducted in Brazilian state capitals in the same year of this study (30.8%). Belo Horizonte was the capital with the highest frequency (35.3%).^e^ This difference could be attributed to the location of the study: PeNSE surveys were completed in the school environment, which may overestimate bullying frequency, whereas this study conducted in home could have underestimated the frequency. The time period associated with the questions could be another factor. The PeNSE study investigated bullying within the last 30 days, whereas the Beagá Health Study asked about bullying during the participant’s lifetime. Asking about the violence suffered only in the previous month may have overestimated the prevalence of bullying because adolescents could have reported cases not considered as bullying (because of the lack of repetition). In addition, the prevalence found in this study is part of the PeNSE study, suggesting that this difference might be due to differences in sample size. Moreover, PeNSE, conducted in 2012 pointed out increased prevalence in the Brazilian capitals between 2009 and 2012.^15^ ^,^ ^f^

The school as the principal site of bullying supports previous studies undertaken in school settings. However, due to the nature of the household setting, this study tested different locations of bullying others than school. The setting for this type of aggression differed in relation to age: younger adolescents tended to report bullying in the school environment, whereas other locations were primarily cited by older teens (17 years old). This difference could be due to lifestyle differences among age groups. Older adolescents may be inserted in other social institutions, such as the workplace. Also, Brazilian adolescents typically finish high school at 16 years old. A Canadian report on adolescents and adolescent health that considered many health issues facing this population, including bullying, has indicated the necessity of studying bullying beyond the school setting. Although urbanization and technological developments favor the occurrence of bullying in other locations, studies consistently show that this type of violence occurs at the school and its proximities.^15^ ^,^ ^22^ The school is a fundamental part of adolescent life since most of their days are spent there. For Lisboa and Koller,^13^ most bullying incidents occur at school because this is the principal microsystem for peer interactions.

Although some studies suggest that bullying occurs mostly among younger adolescents^i^ this study showed a slightly higher bullying prevalence among older adolescents. Large confidence intervals, due to the small sample of older adolescents, contributed to a lack of statistical significance. However, the violence experienced in childhood and teenage years can be repeated in adulthood and, perhaps, these data can help us understand the violence characterized as bullying at workplace in adult life, as presented in recent national^5^ and international studies.^23^

No significant differences between reports of bullying among students of public *versus* private schools was found. These findings support the results of a comprehensive Brazilian study,^14^ showing that bullying occurs in the school setting regardless of its location, size, grade levels or affiliation.^c^ These results suggest that bullying is not limited to specific communities in today’s society.

The prevalence of bullying was higher among adolescents who reported involvement in physical aggression, drug-use, drunkenness, and current tobacco consumption. Literature describes the association between alcohol, tobacco, and bullying.^16^ The World Health Organization highlights the strong association between an increase in violence and the consumption of alcohol and other drugs, which constitute a major public health challenge. The impact of this association is relevant, leading to serious health and familial consequences, and weakening social networks.^j^ Reports of bullying were more frequent among adolescents who reported poor family perceptions. Smith^20^ states that among the risk factors for bullying are family issues, especially those resulting in violence and related to an environment lacking in affection. According to Debarbieux and Blaya,^8^ the home environment and the relationships established in this context can favor aggressive behaviors that characterize bullying. The home environment is generally labeled as “difficult” or “disturbing” in most cases, and frequently children who exhibit aggressive behaviors at school have been subjected to domestic violence.^8^

Bullying was also related to poor social perceptions. The victim is typically unsociable, insecure, passive, withdrawn, and has low self-esteem.^7^ Maybe these particular characteristics can influence the victim’s choice. The absence of reciprocity, a vital characteristic of bullying, can explain the preference of the aggressor for the most fragile victims. According to Debarbieux and Blaya,^8^ victim difficulties in interacting with peers, physical disability and differences in physical appearance are factors that predispose the victim to intimidation. Furthermore, bullying can cause loss of interest in school, poor academic performance, and damaged social relationships.^8^ However, due to the similarity between risk factors for and consequences of bullying, the characteristics of social interactions were not included in the adjusted analysis, although associated with bullying in univariate analysis and interrelated in exploratory analysis.

The prevalence of bullying was higher among those reporting life dissatisfaction and poor psychological well-being. In a study with 16,210 children between eight and 18 years, and their parents in 11 European countries, psychological problems were associated with bullying in almost all of them.^1^ Studies suggest that bullying may lead to both behavioral and emotional problems.^3^

Bullying was most frequent among adolescents whose household heads were more educated. As educational attainment of the household head can be considered a marker of socioeconomic status, and these adolescents may have a greater awareness of bullying and more contact with technologies that facilitate other forms of bullying, such as cyberbullying, not specifically addressed in this study.

Geographic areas with less vulnerability, as measured by the IVS, had a higher prevalence of bullying, which was also seen among adolescents who were afraid of walking around their neighborhood. These characteristics could also serve as indicators of socioeconomic status. Bullying was reported more among adolescents with higher socioeconomic status. The higher prevalence among the more affluent could be attributed to greater knowledge of the issue and access to media, which encourages reporting violence, contributes to the culture of fear, and even trivializes bullying by excessively exposing the adolescent to the theme; on the other hand, other manifestations of violence may interfere with the perception of bullying, making it seem less important in comparison for those adolescents with lower socioeconomic status.

The cross-sectional design of this study does not allow causal inference. Other limitations include: possible underreporting information bias because of household application of the questionnaire; cross-cultural differences, varying interpretations of bullying; no temporal specifications for the question used and, focus on the object of study (victim, aggressor, or both). The greatest limitation could be the lack of a qualitative approach for deeper exploration of bullying motives. However, the adolescents had great ease with the questionnaires, the self-application was meant to provide privacy and the sample size was sufficient, with a power of 85.0% assuming a 95% confidence interval.

Despite these limitations, the study showed a high prevalence of bullying and unveiled self-perception, family and home environment risk factors associated with this contemporary type of violence among adolescents. It allows advancing knowledge on bullying, especially in urban centers of developing countries.

Studies on bullying are relatively recent and present challenges. The finding that approximately 1/4 of adolescents in an urban center reported being victims of bullying suggests that many children and adolescents are at risk of suffering from regular violence from peers. Individual and contextual characteristics found to be risk factors for bullying were obtained by questions that reflected the perceptions of the participant. Studies are needed to further explore gender differences and to explore bullying with a qualitative perspective. Our findings support national and international studies pointing to individual characteristics, peer relations, and familial influences as risk factors of bullying.^17^ Our study highlights, in an unprecedented manner, the importance of within and between household variables such parents’ relationship, self perceptions of neighborhood violence, and contextual neighborhood variable based on the IVS. Territory can be understood as a result of historical, environmental, and social factors that promote the production of health-related events. Under the urban health perspective, context indicators in epidemiological studies contribute to the understanding of neighborhood factors and possible social determinants of health, such as violence.

This theme requires dialogue between the family and school, in addition to policies focusing on multidisciplinary interventions for the reduction of bullying. Peer bullying reflects society’s tolerance to urban violence. Studies to better understand bullying become important for supporting effective public health measures for the reduction of bullying and its consequences.

## Footnotes

a Olweus D. Acoso escolar: hechos y medidas de intervenciones. Ponencia presentada en el Encuentro Europeo por la Convivencia; 2007 [cited 2010 Mar 20]. Available from: http://www.educa.madrid.org/binary/20/DAN_OLWEUS.pdf

b United Nations Children’s Fund. Child poverty in perspective: an overview of child well-being in rich countries. Florence, Italy: UNICEF Innocenti Research Centre; 2007 [cited 2017 Sept 9]. (Report Card; 7). Available from: http://www.UNICEF-irc.org/publications/pdf/rc7_eng.pdf

c Lopes Neto AA, Saavredra LH. Diga não para o bullying – programa de redução do comportamento agressivo entre estudantes. Rio de Janeiro (RJ): Associação Brasileira Multiprofissional de Proteção à Infância e à Adolescência; 2003.

d The Chief Public Health Officer’s. Report on the State of Public Health in Canada, 2011: Youth and Young Adults – Life in Transition. Ottawa; 2011 [cited 2012 Feb 12]. Available from: http://publichealth.gc.ca/CPHOreport

e Instituto Brasileiro de Geografia e Estatística. Pesquisa Nacional de Saúde do Escolar. Rio de Janeiro (RJ); 2009 [cited 2013 Apr 15]. Available from: http://www.ibge.gov.br/home/estatistica/populacao/pense/pense.pdf

f Instituto Brasileiro de Geografia e Estatística. Pesquisa Nacional de Saúde do Escolar. Rio de Janeiro (RJ); 2012 [cited 2014 Oct 21]. Available from: http://www.ibge.gov.br/home/estatistica/populacao/pense/2012/pense_2012.pdf

g Instituto Brasileiro de Geografia e Estatística. Sinopse do Censo Demográfico 2010. Rio de Janeiro (RJ); 2011.

h Universidade Federal de Pelotas. Centro de Pesquisas Epidemiológicas. Estudo da Coorte de nascimentos de 1993 em Pelotas/RS. Pelotas (RS): Faculdade de Medicina, UFPel; 1993 [cited 2013 Apr 13]. Available from: http://www.epidemio-ufpel.org.br/site/content/coorte_1993/index.php

i Chantala K, Tabor J. Strategies to perform a design-based analysis using the add health data. Chapel Hill: University of North Carolina; 1999 [cited 2009 Mar 12]. Available from: http://www.cpc.unc.edu/projects/addhealth/data/using/guides/weight1.pdf

j World Health Organization. Interpessoal violence and illicit drugs. Geneva; 2009 [cited 2011 Apr 12]. Available from: http://www.who.int/topics/alcohol_drinking/en/
